# Current approach in diagnosis and management of anterior uveitis

**DOI:** 10.4103/0301-4738.58468

**Published:** 2010

**Authors:** Rupesh V Agrawal, Somasheila Murthy, Virender Sangwan, Jyotirmay Biswas

**Affiliations:** L. V. Prasad Eye Institute, Kallam Anji Reddy Campus, Hyderabad, India; 1 Medical Research Foundation, Sankara Netralaya, Chennai, India

**Keywords:** Anterior uveitis, iridocyclitis, iritis, uveitis

## Abstract

Uveitis is composed of a diverse group of disease entities, which in total has been estimated to cause approximately 10% of blindness. Uveitis is broadly classified into anterior, intermediate, posterior and panuveitis based on the anatomical involvement of the eye. Anterior uveitis is, however, the commonest form of uveitis with varying incidences reported in worldwide literature. Anterior uveitis can be very benign to present with but often can lead to severe morbidity if not treated appropriately. The present article will assist ophthalmologists in accurately diagnosing anterior uveitis, improving the quality of care rendered to patients with anterior uveitis, minimizing the adverse effects of anterior uveitis, developing a decision-making strategy for management of patients at risk of permanent visual loss from anterior uveitis, informing and educating patients and other healthcare practitioners about the visual complications, risk factors, and treatment options associated with anterior uveitis.

Anterior uveitis is the commonest form of intraocular inflammation with a varying incidence in the general population of various countries around the world.[1] The potential severe consequences of recurrent or untreated anterior uveitis are probably underestimated.[2] Anatomically, anterior uveitis involves inflammation of the iris alone (iritis), anterior part of ciliary body (anterior cyclitis) or both structures (iridocyclitis).[3] It is commoner than posterior segment inflammation and is generally less sight-threatening and less serious, especially if treated early. Anterior uveitis normally causes reduction in the vision during the acute stage but it is the sequelae of anterior uveitis which can have long-lasting impact. The purpose of the present article is to highlight the diagnosis of anterior uveitis and its various subtypes and to outline management strategies for each. Detailed medlars search was carried out to review the articles and case reports on anterior uveitis. The methods used to collect/select evidence were hand-searches of published literature (primary sources) and searches of electronic databases. The current guidelines for diagnosis and management of anterior uveitis are based on the literature search using the National Library of Medicine's Medline database and the Vision Net database.[3–19]

The uveitis was classified in different ways. Classification based on the duration of uveitis was based on Standardization of Uveitis Nomenclature (SUN) criteria[3] in which anterior uveitis was classified as limited (less than or equal to three months duration) and persistent (more than three months). Based on the course of uveitis, anterior uveitis was classified as acute anterior uveitis with episodes of sudden onset and limited duration, recurrent anterior uveitis with repeated episodes separated by periods of inactivity without treatment ≥ three months in duration, and chronic uveitis which persists and relapses in less than three months after discontinuing treatment. Based on etiology[18] anterior uveitis was classified as infectious (such as viral, bacterial, fungal or protozoal), autoimmune with only ocular involvement or with systemic disease association or presenting as masquerade syndrome. Anterior uveitis with other etiologies can be post-traumatic, post-surgical, lens-induced and drug-induced. Pathologically anterior uveitis was classified as granulomatous or non-granulomatous based on the nature of keratic precipitates.[4]

## Clinical features[4–17]

Anterior uveitis can present with acute, chronic or recurrent attacks. Anterior uveitis is the commonest type of intraocular inflammation and commonly presents as unilateral presentation with pain or photophobia, circumlimbal redness and anterior chamber cells and flare. Patients with anterior uveitis usually complain of pain, redness, blurred vision, and photophobia, watering.^15^ Most of the patients would have had repeated attacks and would have sought consultation with multiple ophthalmologists and would have used topical and/or systemic medications on and off. Blurring of vision, which is perhaps the commonest symptom, is caused by turbidity of the aqueous. Photophobia is commonly due to ciliary muscle spasm but anterior chamber cellular infiltration, corneal epithelial edema and pupillary muscle involvement can also contribute. Varying degree of pain seen in anterior uveitis can be attributed to ciliary muscle spasm. It is usually a dull aching type of pain or a throbbing sensation. Severe pain can be associated with raised intraocular pressures.[15,16] The common clinical signs with which a patient of anterior uveitis can present are:[4–17]

**Figure 1:**
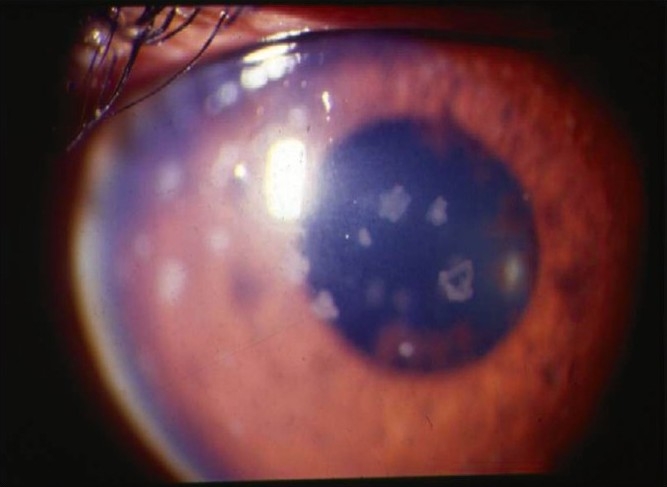
Slit-lamp photograph showing large old keratic precipitates

**Figure 2:**
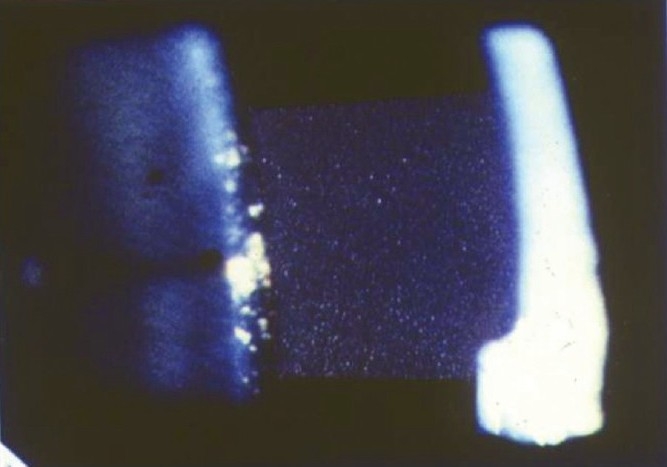
High magnification slit-beam photograph 3×1 mm in size in dark room showing presence of cells and flare

**Figure 3:**
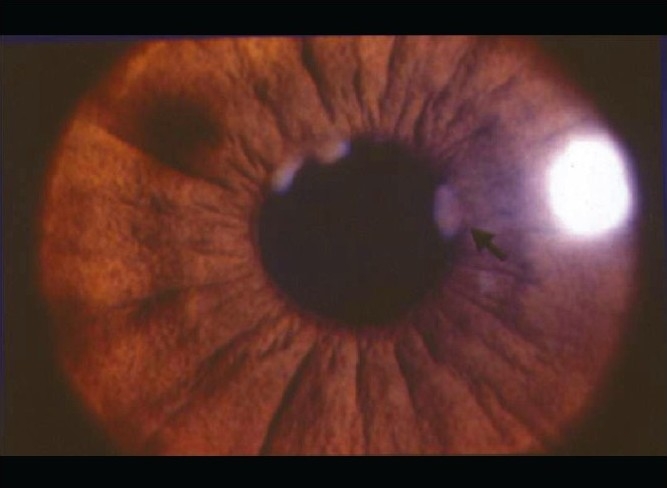
Koeppe Nodules - Nodules present at the papillary margin

**Figure 4:**
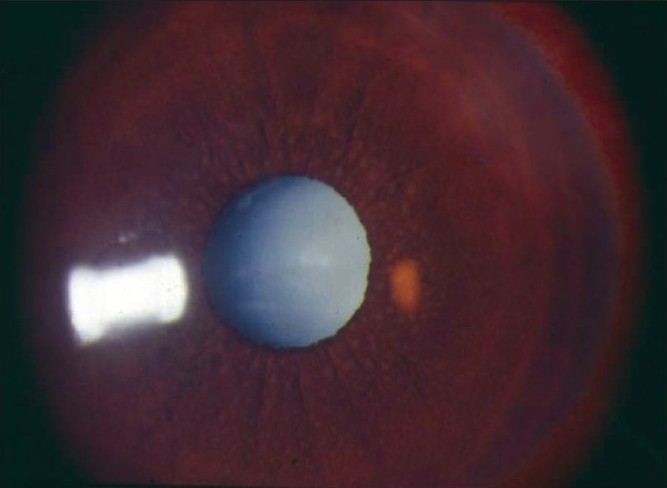
Bussaca Nodules - Nodules present on the iris surface

**Figure 5:**
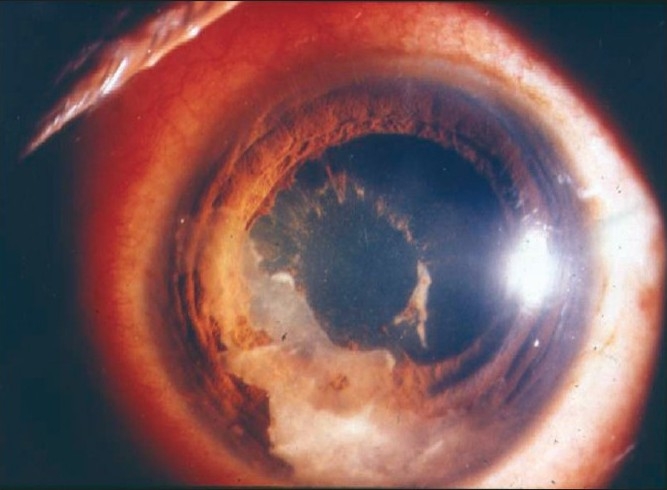
Fibrinous membrane in the anterior chamber between cornea and iris

**Figure 6:**
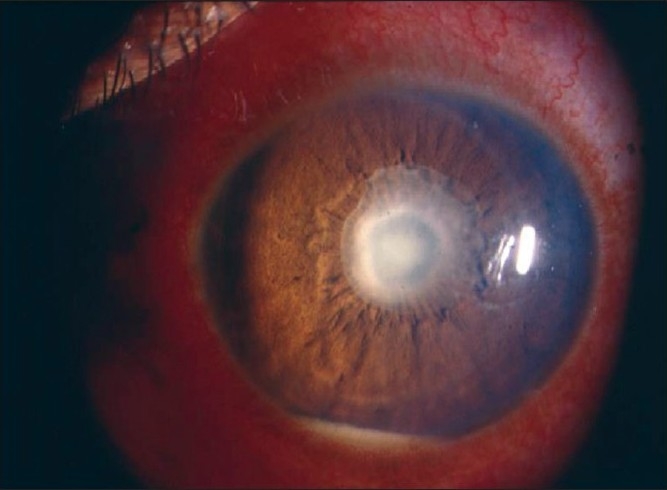
Pupillary membrane with hypopyon

**Figure 7:**
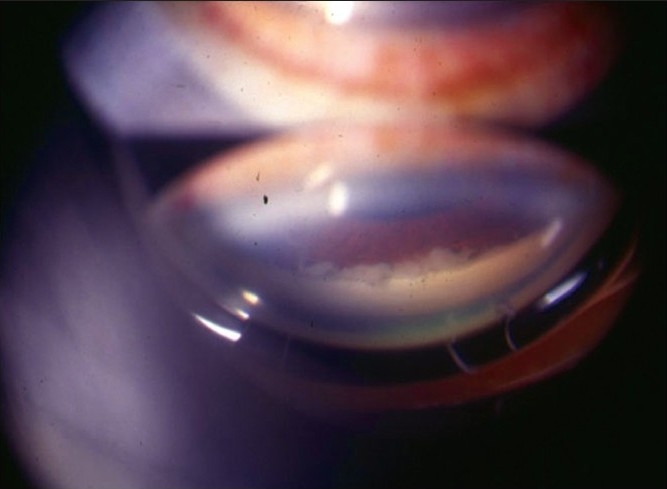
Gonioscopy showing fibrillar deposit in angle

Nil to varying degrees of lid edema may be a presenting sign in patients with anterior uveitis. Circumcorneal congestion may be seen due to enlargement of the episcleral vessels in the region of the ciliary body. Keratic precipitates (KPs) are cellular deposits on the corneal endothelium. Fine KPs are presumed to be of non-granulomatous allergic type of inflammation whereas large and mutton fat KPs are associated with granulomatous inflammation [Fig. 1]. Colored or pigmented KPs suggest prior episodes of anterior uveitis. Microscopically, KPs are accumulation of lympho-plasmocytic inflammatory cells, with epitheloid cells seen additionally in granulomatous KPs.[18] Aqueous cells and flare are due to cellular infiltration and protein exudation into the anterior chamber. Aqueous cells are an early and definite sign of active inflammation. The translucence of the aqueous due to its high albumin content is called aqueous flare. It is an indefinite sign of active inflammation [Fig. 2]. Examination of the anterior chamber involves observing with high magnification while directing a small, intense beam obliquely through the aqueous, following relative dark adaptation. Anterior chamber cells and/or flare are visible owing to the Tyndall effect of the bright beam. The grading of cellular reaction in the anterior chamber helps in the assessment of the severity of anterior uveitis [Table 1]. Grading is useful in determining the patients' response to therapy as well as long-term monitoring. Miosis can be due to reflex spasm of the sphincter or due to vascular distension of the iris vessels. Iris nodules are accumulations of leukocytes lying on the anterior iris; Koeppe's nodules are seen at the pupillary margin [Fig. 3] whereas Busaca's nodules are present on the anterior iris stroma [Fig. 4]. The nodules on the surface of the iris need to be differentiated from infected nodules.[18] Posterior synechiae are the adhesions between the anterior lens surface and the iris; posterior synechiae extending for 360° are called seclusio pupillae while occlusio pupillae refer to a membrane obscuring the lens surface; anterior chamber can show fibrinous reaction [Fig. 5], hypopyon, pupillary membrane with hypopyon [Fig. 6] and hyphema. Iris atrophy is associated with chronic iridocyclitis and occurs due to ischemia. Neovascularization can occur on the iris stroma or in the anterior chamber angle, which may lead eventually to neovascular glaucoma. Anterior vitreous cells are far exceeded by aqueous cells in iritis, whereas in iridocyclitis with intermediate uveitis the cells are distributed equally between the two compartments. Complicated cataract occurs due to thickened lens capsule due to posterior synechiae or altered membrane permeability. Inflammation can result in either increased or decreased intraocular pressure. Acute attack of anterior uveitis with severe anterior chamber inflammation can lead to increase in intraocular pressure and is most commonly seen in viral keratouveitis or Posner Schlosman syndrome. However, idiopathic anterior uveitis can also present with raised intraocular pressure. Fuchs' heterochromic iridiocyclitis is known to be associated with intractable open-angle glaucoma in late stages and the patients need to be counselled about the same. Severe inflammation of the ciliary body may lead to decreased aqueous production and subsequent fall in intraocular pressure may be a result of the inflammation itself, sequelae of inflammation, or because of the steroid treatment. Presence of cyclitic membrane over ciliary body in cases with chronic or recurrent intermediate uveitis with spillover anterior uveitis also leads to severe hypotony. In active inflammation, raised intraocular pressure can be due to associated trabeculitis or can be due to secondary angle closure.[15,16] Though associated trabeculitis is not typical of any specific anterior uveitis entity it can be commonly seen in cases with viral keratouveitis. Gonioscopy would reveal gonio-synechiae or neovascularization in angles, and the angles could be open or closed angles depending on the stage of uveitis [Fig. 7]. The following features should be looked on fundus examination such as disc edema and hyperemia, vascular sheathing, perivascular exudates, cystoid macular edema, retinitis, chorodial infiltrates, retinal detachment, pars plana exudates or snow banking. Presence of positive findings on posterior segment examination indicates anterior uveitis as a part of panuveitis (e.g. sarcoidosis, tuberculosis, Vogt Koyanagi Harada Disease, sympathetic ophthalmia) or as spillover uveitis in cases with intermediate uveitis.

**Table 1:** SUN Working Grouping Grading of cells and flare

AC Flare	Grade	Cells/ field
0-None	0-	<1
	0.5	1-5
1+ Faint	1+	6–15
2+ Moderate (Iris and lens details clear)	2+	16–25
3+ Marked (Iris and lens details clear)	3+	26 –50
4+ Intense (Fixed and plastic aqueous)	4+	50+

## Systematic approach to a patient with anterior uveitis

Detailed systematic approach to a patient with anterior uveitis will comprise the following four-pronged approach:

1. Detailed history
2. Ocular examination
3. Physical examination – As and when required
4. Ancillary investigations - As and when required

### 

#### 1. Detailed History

As with all branches of medicine, a complete history is crucial to the diagnosis and management. History of present illness in terms of onset and progression of symptoms, course and treatment received with special emphasis on corticosteroids therapy should be ascertained. Past ocular history should elicit recurrent attacks of uveitis and previous response to treatment. Detailed systemic history forms a very important part in the management of uveitis patients. Apart from a review of systems, the social history for pets, dietary history, sexual and drug history should be obtained in detail.

#### 2. Ocular Examination

A careful examination of both the anterior and posterior segments is of utmost importance.

#### 3. Physical Examination

**Figure 8:**
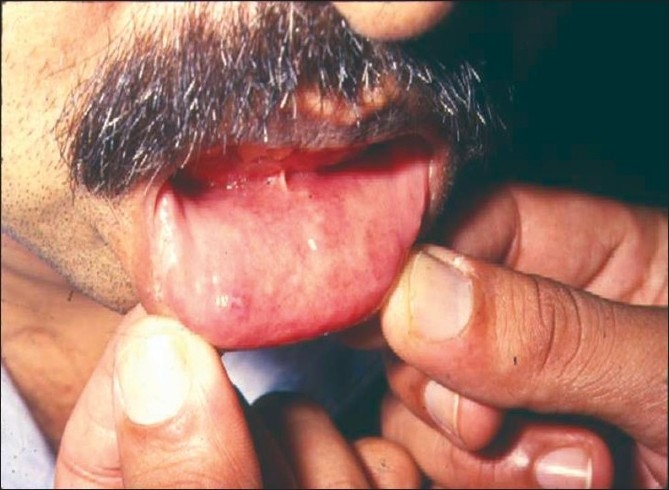
Oral ulceration seen in Behcet's disease (painful ulcers as against painless ulceration seen in Reiter's syndrome)

**Figure 9:**
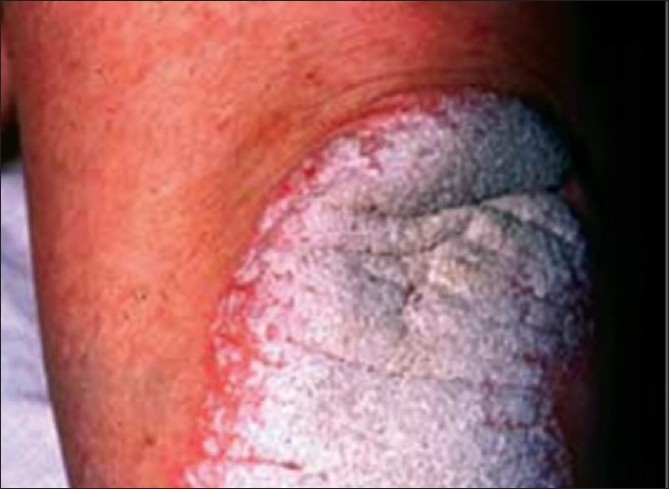
Skin lesions seen in psoriasis

**Figure 10:**
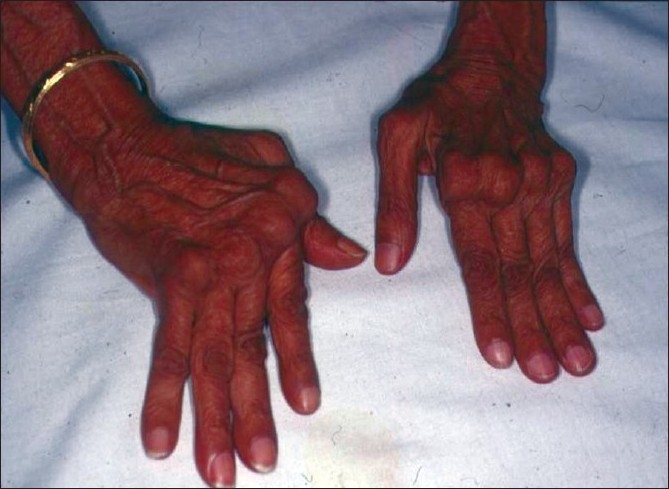
Joint deformity seen in rheumatoid arthritis

Relevant physical examination by the internist to rule out any other systemic association is important in cases suspected to have systemic association based on the history. Anterior uveitis can be associated with dermatological, respiratory, rheumatologic, genital, gastrointestinal or neurological findings.[20–29] Some of the systemic associations are as shown in Figs. 8–10.

#### 4. Ancillary investigations: Tailored Approach

**Ocular investigations:** These would be sometimes required, including B-scan ultrasonography, fluorescein angiography, and optical coherence tomography for the assessment of posterior segment. Ultrasound biomicroscopy would be useful in cases with small pupils and hypotony to assess the status of ciliary body and presence of cyclitic membranes.[30]

**Laboratory investigations:** A rank ordered test of possible diagnoses should be made from the history and examination. Then, investigations should be ‘tailored’ to confirm the diagnosis.[31–33] Investigation of a patient with a first episode of anterior uveitis will depend mainly on history and examination. The investigation should be targeted to rule out associated systemic disease and infectious cause of uveitis [Table 2].[31–33]

**Table 2:** Suggested laboratory tests, X-ray studies, consults/referrals or other tests to isolate systemic causes of anterior uveitis

Disease suggested by history and examination	Lab tests	X-Ray studies	Consult/ referral	Other tests
Ankylosing spondylitis	↑ ESR, (+)HLA-B27	Sacroiliac X-rays	Rheumatologist	
Inflammatory bowel disease	HLA B27+ve		Gastroenterologist	
Reiter's syndrome	↑ ESR, (+)HLA-B27	Joint X-rays	Urologist, Rheumatologist	Cultures – conjunctiva, urethra, prostrate
Psoriatic arthritis	HLA B27 +ve	Rheumatologist, Dermatologist	
Herpes	Clinical diagnosis	Dermatologist	
Behcet's disease	HLA B51 +ve	Internist or Rheumatologist	
Lyme disease	ELISA or Lyme immunofluorescent assay		Internist, rheumatologist	
Juvenile rheumatoid arthritis	↑ ESR, (+)ANA, (-)Rheumatoid factor	Joint X-rays	Rheumatologist or pediatrician	
Sarcoidosis	↑ Angiotensin converting enzyme (ACE)	Chest X-ray	Internist	
Syphilis	(+)RPR or VDRL; FTA-ABS or MHATP		Internist	
Tuberculosis		Chest X-ray	Internist	Purified
				protein
				derivative
				(PPD) skin test

ESR – Erythrocyte Sedimentation Rate, HLA – Human Leukocyte Antigen, PPD _ Purified Protein Derivative, ANA – Antinuclear antibody, VDRL – Venereal Disease Research Laboratory, FTA-ABS – Fluorescent treponmenal antibody test

* Adapted from Cullen RD, Chang B, editors. The Wills eye manual. Philadelphia: JB Lippincott, 1994. p. 354-5.

Investigations are not required in first episode of non-granulomatous anterior uveitis or in Herpes zoster ophthalmicus-associated uveitis and Fuchs' heterochromic iridocyclitis.

In other cases, investigations which may be advised:

- Complete Blood Count:-Baseline, Leucocytosis in infectious etiology
- Erythrocyte sedimentation rate (ESR): Nonspecific indication of a systemic disease.
- Mantoux test: Nonspecific test. Indicates prior exposure to tubercle bacilli.
- Venereal disease research laboratory test (VDRL): Nonspecific screening test for syphilis.
- Treponema pallidum hemagglutination test (TPHA): Highly specific for syphilis.
- Human leukocyte antigen (HLA) B27: To be done in patients with recurrent attacks of anterior uveitis, positivity of HLA B27 helps the clinician in counselling the patient for recurrent attacks of uveitis which are more severe and more frequent in nature.
- Antinuclear Antibodies: Collagen vascular disease.
- Serum Angiotensin Converting Enzyme Assay Active sarcoidosis. However it can be normal in patients with sarcoidosis and it can be physiologically elevated in chronic smokers and in children. Interpretation of serum ACE test has to be done in conjunction with clinical findings.
- Chest X-ray: Tuberculosis, Calcified hilar lymphadenopathy in Sarcoidosis;
- Sacroiliac joint X-ray: Ankylosing Spondylitis
- High resolution CT scan chest: Sarcoidosis
- Tridot analysis for HIV: Any unusual presentation of uveitis.

The above listed tests have to be done on a tailored basis and not all the tests are usually required in all patients with anterior uveitis. Similarly, in selected cases with keratouveitis, the aqueous humor study for Herpes Simplex Virus or other viruses can be carried out using polymerase chain reaction (PCR) study.

## Specific disease entities in anterior uveitis

### 

#### A. Non-infectious cause

1. Idiopathic: Isolated inflammation of the anterior segment. Non-granulomatous and may have seasonal variation. Accounts for 50% of cases.

- Ankylosing spondylitis (HLA B27) related iridocyclitis: More common in males, and has a younger age of onset. Can be associated with systemic features.[22–24] It is believed to be triggered by gram-negative bacilli, which interact with HLA B27.[22–24]
  Ocular features: unilateral, recurrent, acute iridocyclitis. Ocular features do not correlate with the severity of systemic disease.
  Systemic features: sacroilitis with morning stiffness that improves with exercise.
- Reiter's syndrome: Young males in the age group of 15-35 years. The classical triad consists of arthritis, urethritis and conjunctivitis. Acute non-granulomatous, recurrent uveitis is the hallmark. Other ocular features are mucopurulent conjunctivitis, subepithelial keratitis. Other systemic features are keratoderma blenorrhagica, balanitis, and aphthous stomatitis.[21,25]
- Inflammatory bowel disease: Crohn's disease, ulcerative colitis and Whipple's disease may present with acute recurrent iridocyclitis.[21,25]
- Psoriatic arthritis: Patients have recurrent acute iridocyclitis. Systemic features: psoriatic skin changes and seronegative, erosive arthritis in the small joints.[25,26]

### 2. Juvenile idiopathic arthritis-associated uveitis:[27]

This can be classified into those with systemic onset, also known as Still's disease (these patients rarely have ocular manifestations); polyarticular onset with five or more joints involved (7-14% patients develop acute anterior uveitis), and pauciarticular onset with involvement of less than five joints. This last subtype is commonly associated with anterior uveitis. Subgroups are Type I-ANA positive: presents with chronic iridocyclitis in young girls and Type II recurrent acute anterior uveitis in older boys; 75% are HLA B27 positive.[27] Most juvenile idiopathic arthritis (JIA)-associated uveitis presents in the pediatric age group and more commonly in girls. The uveitis is not associated with much cirucmcorneal congestion and hence it is often termed as uveitis with white eye. Complications in the form of complicated cataract and band-shaped keratopathy are common in this uveitic entity mainly because of its late presentation as it's ignored by the patient and parents because of lack of associated redness and pain.

### 3. Lens-induced uveitis:

The uveitis secondary to lens can present as phacoanaphylactic endophthalmitis which is an immune complex-mediated reaction to lens protein. It presents as a sterile granulomatous uveitis after cataract surgery or after traumatic rupture of the lens capsule. Lens-induced uveitis can be phacogenic uveitis which occurs after exposure to liberated lens protein. It can be also seen along with phacolytic glaucoma in hypermature cataracts in which leakage of lens protein occurs through an intact capsule. There is blockage of the trabecular meshwork by the denatured lens protein leading to secondary glaucoma.[16]

### 4. Intraocular lens (IOL)-related uveitis:

Can occur by various mechanisms: mechanical irritation – due to excessive manipulation during surgery; chaffing of the iris by the IOL, Uveitis-Glaucoma- Hyphema (UGH) syndrome is seen in rigid type of anterior chamber (AC) IOL whose footplates erode into the iris root. It can also be secondary to foreign-body reaction to IOL polymers or can present as toxic lens syndrome which is a sterile uveitis due to IOL impurities, metal haptics, poor finish of optic / optic-haptic junction (toxic anterior segment syndrome).[16]

### 5. Fuchs heterochromic iridocyclitis:

Characterized by idiopathic non-granulomatous anterior uveitis. It is of insidious onset and recurrent in nature. It's commonly seen in young females, mostly unilateral, and patients commonly present with blurring of vision. As the uveitis is mild, it's usually not associated with circumcorneal congestion. Classical presentation of uveitis in Fuchs heterochromic iridocyclitis is occasional to 1+ anterior chamber cells, aqueous flare of 1+ to 2+, diffuse white stellate keratic precipitates on the endothelium which are non-pigmented non-confluent in nature. Absence of posterior synechiae is the pathognomic sign of Fuchs heterochromic iridocyclitis. Most patients present with posterior subcapsular cataract in early stages and with total white cataract in late stages. Intraocular pressure (IOP) is usually normal in early stages but in late stages there is increase in IOP which is usually refractory to medical and even to surgical therapy. There can be presence of vitreous haze in cases with Fuchs heterochromic iridocyclitis. Most cases with Fuchs heterochromic iridocyclitis do not require any topical steroids to control inflammation as there is very mild uveitis and most commonly aqueous flare. Vitreous haze also does not require any systemic steroid treatment. Outcome of cataract surgery in Fuchs heterochromic iridocyclitis is usually good. Heterochromia iridium is the hallmark of the disease due to iris stromal atrophy.[34]

### 6. Traumatic iridocyclitis:

This may be due to blunt trauma or it may be post-surgical. The uveitis is due to the breakdown of the blood-aqueous barrier. It is self-limiting in nature.[16]

### B. Uveitis related to infectious agent:

#### 1. Herpetic keratouveitis

Usually presents with insidious onset of blurred vision associated with pain and redness of the eye. There is presence of circumscribed corneal edema with fine pigmented KPs present on the endothelium posterior to the area of stromal edema, reduced corneal sensation, AC reaction of any grade, presence or absence of posterior synechiae. The diagnosis is clinical and extensive oral and topical antiviral therapy is required along with topical steroids and cycloplegics. IOP is found to be usually raised in patients with viral keratouveitis and hence antiglaucoma medications are often required in the management of viral keratouveitis.

#### 2. Tuberculosis

Presence of mutton fat KPs and Bussaca nodules and posterior synechiae points towards underlying chronic granulomatous disease such as sarcoidosis or tuberculosis. Ocular tuberculosis need not be associated with pulmonary Koch's.

#### 3. Varicella Zoster

Herpes zoster ophthalmicus is reactivation of the *Varicella Zoster* virus. It can occur with or without trigeminal neuralgia, presenting with severe pain along the distribution of the trigeminal nerve in cases of neuralgia. It's usually associated with involvement of the tip of the nose with *varicella zoster* vesicle at the tip of the nose (Hutchison's sign). Ocular findings include unilateral non-granulomatous or granulomatous iritis or iridocyclitis, anterior and posterior syenchiae, increased IOP, sectoral iris atrophy which may be complicated by cataract formation.[8,11–14]

## Management of anterior uveitis:[35–38]

Anterior uveitis can generally be managed by medical therapy and requires surgical intervention only if structural complications supervene and those can be either secondary glaucoma or secondary cataract. The general goals of medical management are:

- Relief of pain and photophobia
- Elimination of inflammation
- Prevention of structural complications such as synechiae, secondary cataract and glaucoma
- Preservation or restoration of good visual function.

However, one needs to remember the potential side-effects and long-term iatrogenic complications secondary to the use of steroid therapy. The risk-benefit ratio should be analyzed on a case-to-case basis.

Noninfectious anterior uveitis is treated using the following drugs.[35–38]

### A) Corticosteroids

Corticosteroids are the drug of choice in the treatment of anterior uveitis. Steroids act by modifying and decreasing the inflammatory response in the eye. They inhibit both the cycloxygenase pathways of inflammatory response. Topical corticosteroids if administered as frequent doses, can achieve adequate therapeutic levels in the anterior chamber. However, if topical alone is inadequate, periocular and systemic administrative routes should be considered.

Topical drops are the commonest and easiest method with the least side-effects. A number of topical ophthalmic corticosteroids are available:[35–38]

- Prednisolone acetate 0.125% and 1%
- Betamethasone 1%
- Dexamethasone sodium phosphate 0.1% (also available in 0.05% ointment form)
- Fluorometholone 0.1% and 0.25% (also available in 0.1% ointment form)
- Loteprednol
- Rimexolone 1%

The choice of topical steroid should be based on the severity of uveitis; in cases with severe AC reaction topical steroid with strong potency such as prednisolone acetate should be preferred whereas in cases with mild anterior uveitis weak topical steroid such as betamethasone or dexamethasone can be applied. In steroid responders one should try and avoid steroid as far as possible and can use topical non-steroidal anti-inflammatory drugs (NSAIDs) like flubriprofen or weak steroids or steroids with least propensity to raise IOP such as rimexolone 1%.

Periocular injection is indicated where maximum concentration of the drug is required for a longer time with minimal side-effects. The drugs which can be considered for periocular injections are dexamethasone or triamcinolone acetonide. Posterior subtenon's injection is recommended in intermediate and posterior uveitis.

Systemic corticosteroids are indicated when the anterior uveitis is not responding to topical drugs alone or if the disease is recurrent and bilateral. If there is any component of posterior uveitis, one may need to start oral corticosteroids early itself. The guidelines for oral corticosteroids therapy are:

- Use enough, soon enough, often enough, and long enough to secure the desired results.
- Start with high dose and taper according to the clinical response.
- Suppress inflammation till the pathogenic effect ends.
- The dose of steroid should be planned but in accordance to the response of the disease.

The level of aggression in terms of treatment is based on the putative diagnosis and the clinical manifestations. Traumatic iritis does not require extensive treatment whereas juvenile rheumatoid arthritis requires more aggressive and more prolonged treatment,[38] the further decision regarding the aggressiveness of therapy should also be based on the degree of inflammation, duration of inflammation, history of previous uveitis and how was the response to treatment, risk of structural damage and response to initial treatment.

### B) Mydriatics/cycloplegics:

These are given as supportive measures. They cause paresis of the iris and ciliary muscle and keep the pupils mobile, thereby preventing the formation of synechiae. A short-acting mydriatic/cycloplegic preparation is usually preferred. All cycloplegic agents are cholinergic antagonists which work by blocking neurotransmission at the receptor site of the iris sphincter and ciliary muscle. Cycloplegics serve three purposes in the treatment of anterior uveitis:

- To relieve pain by immobilizing the iris
- To prevent adhesion of the iris to the anterior lens capsule (posterior synechia), which can lead to iris bombe and elevated IOP
- To stabilize the blood-aqueous barrier and help prevent further protein leakage (flare).
- Cycloplegic agents useful in treating anterior uveitis are:
- Atropine, 0.5%, 1%, 2%
- Homatropine, 2%, 5%
- Cyclopentolate, 0.5%, 1%, 2%.
- Phenylephrine, 2.5%, is an adrenergic agonist that causes dilation by direct stimulation of the iris dilator muscle. Because phenylephrine has neither a cycloplegic nor anti-inflammatory effect and may cause a release of pigment cells into the AC, it is generally not recommended as an initial part of the therapeutic regimen. Phenylephrine may, however, help break recalcitrant posterior synechia.

### C. Non-steroidal anti-inflammatory drugs

These work by inhibition of arachidonic acid metabolism and include drugs such as indomethacin, flurbiprofen and diclofenac sodium. However, when used alone, their efficacy in treating acute intraocular inflammation has not been established.

### D. Immunosuppressive agents:

These are used mainly in corticosteroid-resistant cases or as steroid-sparing agents. They are usually not used in acute anterior uveitis except in a few cases like JIA-associated iridocyclitis and Behcet's disease. The immunosuppressives commonly used are methotrexate or azathioprine. One needs to start immunosuppressive in conjunction with the internist. Patients needs to be educated about the side-effects of immunosuppressives and should be strictly asked for regular blood and systemic examinations as and when required.

## Monitoring response to treatment

Monitoring of response to treatment should include visual acuity and grading of cells and flare. The schedule of follow-up should depend on the severity of initial inflammation, potential for sequelae and type of therapy instilled. Patients should be carefully monitored for side-effects of corticosteroids and immunosuppressives. If at follow-up, the AC reaction is improving, the clinician may continue or reduce medications, depending on the severity of the initial reaction. Cycloplegics may be discontinued when the cellular reaction is subsiding and flare is absent. Chronic anterior uveitis may require long-term use of low-dose topical steroids. When the patient is a steroid responder (i.e., increased IOP), concurrent treatment with a beta blocker is advised unless contraindicated. Once the patient's condition has stabilized, follow-up should be every one to six months. The longer the eye is quiet, the longer can be the interval between follow-up visits.

The patient should be informed about the serious nature of anterior uveitis. Compliance with the therapeutic regimen and keeping all follow-up appointments are essential to achieve the therapeutic goals. The treating ophthalmologist should advise the patient of the potential side-effects of long-term corticosteroid use (i.e., glaucoma and posterior subcapsular cataracts). It is important that this advice be well-documented in the medical record, and the patient should be reminded periodically throughout the course of treatment.

## Treatment of complications

### 

#### 1. Cataract extraction in a patient with uveitis

Guidelines for cataract extraction in a uveitis patient:

- The eye should be quiet for at least three months and preferably six months prior to cataract surgery.
- Preoperative systemic steroids should be administered three days prior to surgery and posterior subtenon's steroid injection 24-28 h prior to complicated cataract surgery. Systemic steroids should be subsequently continued for period of six weeks following cataract surgery in tapering doses.
- Excessive manipulation of iris during surgery should be avoided.
- Extracapsular extraction/ phacoemulsification with meticulous cleaning of cortical material is mandatory. Intracapsular cataract extraction is not recommended as inflammation could spread to the posterior segment.
- IOL implantation in uveitis: Posterior chamber IOL in the capsular bag is recommended. However, implantation of IOL in cases with JIA should be avoided as it can lead to chronic irritable eye.
- Postoperative systemic steroid.
- Lensectomy with pars plana vitrectomy is recommended in cataract patients with juvenile rheumatoid arthritis' pars planitis and lens-induced uveitis.

#### 2. Uveitic glaucoma

Glaucoma associated with uveitis is one of the most difficult complications to address and manage. Medical therapy with topical or systemic antiglaucoma medications is the first step in the treatment of glaucoma-associated uveitis. When medical therapy does not control the IOP and optic nerve or field damage is documented, a surgical procedure becomes mandatory. The use of anti-proliferative agents is almost always mandatory in uveitic glaucoma whereas use of drainage devices can be considered in intractable cases.

#### 3. Cystoid macular edema

Is the commonest complication of intermediate uveitis-associated anterior uveitis. There is presence of a dull foveal reflex and near vision is usually diminished in cases with cystoid macular edema. The clinical diagnosis can be supported by fundus fluorescein angiography findings in the form of petalloid leakage around the fovea or on optical coherence tomography. Topical NSAIDs for a prolonged period or posterior subtenon's steroid injection forms the mainstay of therapy for cystoid macular edema. In cases with chronic cystoid macular edema one may resort to intravitreal steroid injection.

## Summary

Anterior uveitis is a sight-threatening eye condition that must be diagnosed and treated early by ophthalmologists. Due to its association with potentially serious systemic disease and when undetected can cause loss of vision, the importance of awareness about this entity to primary eye care physician is of public health concern. The differential diagnosis of anterior uveitis can be accomplished by a thorough eye examination and physical assessment. If diagnosed and treated on time, anterior uveitis can be treated without long-term sequelae and even if there are associated complications, they can be managed if detected early.
